# Extra-Gonadal and Non-Canonical Effects of FSH in Males

**DOI:** 10.3390/ph16060813

**Published:** 2023-05-30

**Authors:** Matteo Spaziani, Francesco Carlomagno, Marta Tenuta, Franz Sesti, Francesco Angelini, Ilaria Bonaventura, Davide Ferrari, Chiara Tarantino, Marco Fiore, Carla Petrella, Luigi Tarani, Daniele Gianfrilli, Carlotta Pozza

**Affiliations:** 1 Department of Experimental Medicine, Sapienza University of Rome, 00185 Roma, Italy; 2 Institute of Biochemistry and Cell Biology (IBBC-CNR), Department of Sensory Organs, Sapienza University of Rome, 00185 Rome, Italy; 3 Department of Maternal Infantile and Urological Sciences, Sapienza University of Rome, 00185 Roma, Italy

**Keywords:** FSH, extra-gonadal, bone, cardiovascular system, immune system, metabolism, infertility, prostate cancer

## Abstract

Recombinant follicle-stimulating hormone (FSH) is commonly used for the treatment of female infertility and is increasingly being used in males as well, as recommended by notable guidelines. FSH is composed of an α subunit, shared with other hormones, and a β subunit, which confers specificity of biological action by interacting with its surface receptor (FSHR), predominantly located in granulosa and Sertoli cells. However, FSHRs also exist in extra-gonadal tissues, indicating potential effects beyond male fertility. Emerging evidence suggests that FSH may have extra-gonadal effects, including on bone metabolism, where it appears to stimulate bone resorption by binding to specific receptors on osteoclasts. Additionally, higher FSH levels have been associated with worse metabolic and cardiovascular outcomes, suggesting a possible impact on the cardiovascular system. FSH has also been implicated in immune response modulation, as FSHRs are expressed on immune cells and may influence inflammatory response. Furthermore, there is growing interest in the role of FSH in prostate cancer progression. This paper aims to provide a comprehensive analysis of the literature on the extra-gonadal effects of FSH in men, with a focus on the often-conflicting results reported in this field. Despite the contradictory findings, the potential for future development in this area is substantial, and further research is needed to elucidate the mechanisms underlying these effects and their clinical implications.

## 1. Introduction

Follicle-stimulating hormone (FSH) was first identified in 1930. This hormone plays a central role in mammals’ reproduction, and its synthesis is regulated by gonadotropin-releasing hormone (GnRH) pulsatile release, leading to the activation of the hypothalamic–pituitary–gonadal (HPG) axis, first during mini-puberty (lasting, in boys, from the first to the sixth/ninth month of life), and then in puberty [1].

FSH is a heterodimeric glycoprotein hormone made of two subunits (α and β), with a molecular weight of 28–30 kDa, secreted by the anterior pituitary gland. Its structure is shared with other glycoproteic hormones, including luteinizing hormone (LH), thyroid-stimulating hormone (TSH) and human chorionic gonadotropin (hCG). The common α subunit is coupled with the hormone-specific β subunits, and the presence of both is required for biological activity. The FSH β subunit consists of 111 amino acids, conferring specific biologic action and responsible for the interaction with the FSH receptor (FSHR) [2], localized on the surface of target cells, e.g., on the testes and ovaries (granulosa and Sertoli cells). The FSHR belongs to the family of G protein-coupled receptors, (GPCRs) involved in various signaling pathways (i.e., cAMP/PKA, PKC/MAPK, and Ca+/CaMKII) [3,4,5].

FSH plays a pivotal role in reproduction, stimulating antrum formation in secondary follicles, as well as growth and maturation in antral follicles in females, whereas in males FSH is responsible for testicular development and maintenance. In mature gonads, it acts on Sertoli cells, spermatogonia, primary and secondary spermatocytes, and drives spermatogenesis [6].

FSH preparations (urinary, purified, and recombinant) are widely used in the treatment of female infertility, but increasingly it is being used to treat males with hypogonadotropic hypogonadism or idiopathic infertility with inappropriately normal FSH levels.

According to the 2018 European Academy of Andrology (EAA) guidelines on oligo-astheno-teratozoospermia (OAT), FSH treatment can be offered to selected men who are normo-gonadotropic with idiopathic OAT, although with low evidence [7]. The Italian Society of Andrology and Sexual Medicine (SIAMS) recently suggested the use of FSH preparations to increase sperm concentration and motility in infertile normo-gonadotropic men with idiopathic OAT, with moderate evidence grading [8], although this is limited by the relatively high costs and off-label usage in some countries [9]. In hypogonadotropic hypogonadism the use of FSH is more easily recommended [10]. Beyond the well-known effects of FSH on male and female reproductive functions, and precisely because of its increasing usage in the treatment of infertility, the attention has recently focused on the extra-gonadal effects of FSH, along with the possible underlying mechanisms. Recent studies, indeed, have found that FSH receptors also exist in extra-gonadal tissues, such as immune cells (monocytes/macrophages and others) [11] and the vascular endothelium [12]. FSH could, therefore, be involved in other pathological and physiological processes, which are still not completely understood.

Therefore, the aim of this review is to provide an overview of extra-gonadal and non-canonical FSH effects in males, focusing on the main physiological and pathophysiological features.

## 2. Gonadal Effects of FSH

In males, FSH activates Sertoli cell proliferation, first during fetal development, then during mini-puberty, and continuing at puberty, when it induces spermatogenesis, whilst LH induces Leydig cells’ steroidogenesis to produce androgens [13]. In adult life, FSH acts through its receptors on Sertoli cells and germ cells up to the secondary spermatocyte stage, promoting meiosis entry and limiting overall germ cell apoptosis. Testosterone (T) is responsible for the later spermatogenesis stages, comprising spermiogenesis [14]. In this regard, FSH and T independently regulate spermatogenesis in an additive, as well as a synergistic, manner [15], with strong crosstalk with the somatotropic axis [16]: T activates the androgen receptor (AR) in Sertoli cells to initiate the functional responses required for spermatogenesis [17], whereas FSH stimulates Sertoli cells to produce signaling molecules and nutrients to support sperm maturation [3,18]. Moreover, FSH is able to induce LH receptor expression on Leydig cells, therefore influencing steroidogenesis as well [19].

## 3. Extra-Gonadal Effects of FSH

There are currently few studies in the male literature that evaluate the extragonadal effects of FSH. On the other hand, there is increasing interest in considering hormones’ noncanonical effects on different organs and tissues [20]. A lot of evidence comes from animal models and in vitro studies on blood from donors. Existing human clinical trials have been performed primarily in the setting of hypogonadism. An ideal clinical model is represented by patients with previous prostate cancer who underwent androgen deprivation therapy (ADT). Commonly ADT makes use of the following three therapeutic possibilities: (1) GnRH-antagonist (which causes a rapid decrease of FSH levels); (2) GnRH-agonist (responsible for an initial increase of FSH followed by a gradual decrease; (3) bilateral orchiectomy (which leads to very high FSH levels).

A limited number of papers have compared the cortices of patients with hypogonadotropic and hypergonadotropic hypogonadism. Overall, results from in vivo clinical trials in the setting of hypogonadism should be interpreted with caution due to possible confounding of testosterone deficiency or (in the case of hypergonadotropic hypogonadism) of coexisting high LH levels. Finally, a very limited number of randomized control trials (RCTs) have analyzed the extragonadal effects of FSH as a primary outcome after FSH treatment for male infertility.

**Figure 1:**
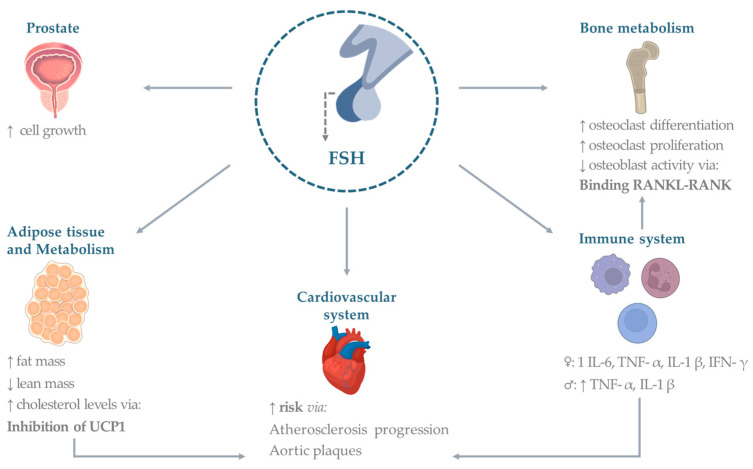
Extragonadal effects of FSH. Established and potential FSH effects on organs and systems. Abbreviations: TNF (tumor necrosis factor), IL (interleukin), UCP (uncoupling protein), IFN (interferon), RANK (Receptor activator of nuclear factor κ B), RANKL (Receptor activator of nuclear factor kappa-Β ligand), FSH (follicle-stimulating hormone). Figures made under Creative Commons License by using the Adobe Stock platform.

Below, we summarize the main evidence divided by individual organs or systems. A summary of the effects is provided in Figure 1.

### 3.1. Bone

Estradiol is known to be the main hormone influencing bone remodeling. Therefore, osteoporosis is conceptually associated with a reduction of estrogens’ action [21]. However, FSH correlates with diminished bone mineral density even years before a decline in estradiol [21,22,23]. Data are still scarce; however, recent in vitro and preclinical studies have shown that FSH stimulates bone resorption by binding to its specific receptors on osteoclasts [24].

In 2006, Sun et al. proved that FSH has a direct role in hypogonadal bone loss, by regulating osteoclast activity in an estrogen-independent manner [25]. In fact, in genetic mice models, either suppressing FSHR (FSHR^−/−^ mice) or its ligand (FSHβ^−/−^ m ice), osteoclastogenic activity did not result in bone loss compared to eugonadal controls (FSHR^+/+^ and FSHβ^+/+^ mice), despite severe hypogonadism, while bone mineral density (BMD) decreased in ovariectomized mice. Interestingly, eugonadal FSHβ haploinsufficient mice (FSHβ^+/−^, with reduced levels of FSH, but normal estrogen levels) showed increased bone mass, suggesting a direct, estrogen-independent action of FSH on bone. In vivo and ex vivo assays also suggested that FSH increases osteoclastogenesis and bone resorption by binding a G_i_2α-protein, coupled to FSHR, on the osteoclasts’ surface, and activating non-canonical signaling, different from that of the ovarian granulosa cells [25]. Similar findings were obtained by blocking the binding of FSH to its receptor, which caused both a reduction in bone resorption and an increase in bone formation by influencing osteoclast and osteoblast activities [26,27,28,29].

In an in vitro study using blood from male and female donors, high FSH concentrations (>50 mIU/mL) were able to promote the development of osteoclasts from circulating precursor cells CD14^+^ by enhancing RANK-L binding to its receptor RANK, a member of the tumor necrosis factor (TNF) receptor superfamily, which drives NF–kB production and is critical to start osteoclast differentiation [11]. Moreover, FSH can also modulate the synthesis of TNF-α [30], alongside interleukin (IL)-1β and IL-6 [31]. These proinflammatory cytokines can stimulate synthesis and differentiation of osteoclasts and inhibit osteoblast activity. Nevertheless, the existing literature in males is still poor and contradictory, and the putative role of FSH as a direct modulator of skeletal physiology remains a matter of debate. According to other findings on animals, no change in bone parameters was observed after one month of intermittent or continuous FSH treatment to skeletally mature male mice, suggesting that high levels of circulating FSH have no effect on bone metabolism in vivo. In support of these results, FSH treatment did not potentiate the osteoclastogenic effect of RANKL to promote osteoclast formation and activity in vitro [32].

Giovanelli and colleagues were the first to compare men with primary and secondary hypogonadism to elucidate the role of FSH in male bone health. Patients with hypergonadotropic hypogonadism showed lower lumbar spine BMD than patients with hypogonadotropic hypogonadism, particularly in trabecular bone, in keeping with what is observed in perimenopausal women and, more generally, in high-turnover osteoporosis [33]. In fact, bone loss was worse in patients affected by Klinefelter syndrome (KS), a condition of raised FSH levels, reflecting the influence of long-term FSH excess, starting from puberty [33]. In line with these findings neither T levels nor AR gene polymorphisms are associated with osteoporosis in patients with KS [34], and testosterone replacement therapy (TRT) in hypogonadal KS men has been shown to solely increase lumbar spine BMD, with no effects on the hip or on bone quality measures, [35] crediting the hypothesis that FSH might play a role in bone health. Furthermore, in a cross-sectional case-control study of 156 men, FSH levels were higher in men with osteoporosis than in healthy controls, data confirmed by the negative correlation between FSH and BMD both at the lumbar spine and femoral neck. More specifically, at multivariate analysis, FSH resulted as a better predictor of bone mass than other gonadal hormones in men [36].

Besides preclinical studies, an association between increased FSH values, increased bone turnover and reduced BMD has been observed in peri- and post-menopausal women [37,38,39]. However, whether FSH has a direct effect on male bone mass is still highly controversial. A long-term follow-up study including men with primary spermatogenic failure, showed that prolonged exposure to high FSH levels has no impact on BMD [40]. Moreover, BMD measured 15 years after the initial fertility assessment, was not different between men with a history of infertility, due to spermatogenic failure, and the control group of men from couples treated with in vitro fertilization (IVF) for female factor infertility. These results are in line with an earlier study by the same research group, showing that BMD in men with idiopathic infertility was similar to fertile men, despite the former group having higher FSH levels than the controls [41]. Furthermore, in an RCT, the administration of GnRH analogs to suppress FSH secretion for 16 weeks, alongside T replacement therapy (TRT), did not affect bone turnover in men, suggesting that FSH does not appear to be a significant regulator of bone in eugonadal men, at least in the short term [42]. However, it must be considered that bone metabolism is normally very slow and that, therefore, the observation time of the study may not have been sufficient to detect changes in bone density.

FSH may also be involved in determining adverse bone effects in the context of CFTR mutation carriers, or in subjects affected by cystic fibrosis [43]. Indeed, CFTR drives FSH-stimulated aromatase expression and activity though increased nuclear soluble adenylyl cyclase activity, regulating estrogen production in humans [44]. As such, decreased CFTR activity would possibly lead to reduced estrogen levels (possibly compensated by an increase in FSH secretion), with consequent bone loss.

### 3.2. Cardiovascular System

Consolidated studies have shown how cardiovascular (CV) risk is associated with low estrogen levels in premenopausal women [45]. More recently, a direct role of FSH on CV risk has been proposed. The Study of Women’s Health Across the Nation (SWAN) correlated FSH levels’ trajectories with atherosclerosis development [46], finding that women experiencing low FSH rise after menopause may be at lower risk of atherosclerosis than those who experience either a medium or a high rise FSH over the transition. Munir et al., in the Assessment of the Transition of Hormonal Evaluation with Non-invasive imaging of Atherosclerosis (ATHENA-CT) study, showed how FSH was directly associated with the number of aortic plaques, using coronary CT angiography and carotid ultrasound [47]. Only a few studies investigated FSH-mediated effects in males. Animal models receiving androgen deprivation therapy (ADT) showed that the largest effects on metabolism, in terms of adiposity and glucose tolerance, and atherosclerosis, were induced, respectively, by orchiectomy, followed by GnRH-agonists and -antagonists, underlying a possible association between FSH levels and the severity of the phenotype [48].

A more recent preclinical study demonstrated that in atherosclerotic mice with apolipoprotein E deficiency (ApoE^−/−^), exogenous FSH supplementation could accelerate atherosclerosis progression, through macrophage activation, increasing the expression and secretion of IL-1β, and, therefore, worsening atherosclerotic lesions [49].

Clinical evidence from six prospective phase 3 RCTs found that patients with pre-existing CV disease, treated with a GnRH antagonist, showed a significantly lower risk of CV events or death compared to patients receiving a GnRH agonist, pointing to a possible role on FSH in these results [50].

Hence, FSH seems to increase CV events, such as heart attacks and stroke, perhaps by promoting unfavorable conditions including inflammation, atherosclerosis, insulin resistance, adipocyte rearrangement and plaque instability [51]. However, some studies contradict these results. Haring et al. found no consistent association of gonadotropin levels and their trajectories with incident clinical CV or all-cause mortality risk in 254 elderly men [52]. Similarly, Kourbanhoussen et al, assessing preoperative FSH levels in 492 men prior to prostatectomy, found that these levels do not appear to be predictors of the long-term incidence of major adverse CV events [53]. Additional research is needed to better understand the potential role of FSH in contributing to CV risk.

### 3.3. Adipose Tissue

A growing body of evidence demonstrates a possible role of FSH in regulating lipid metabolism, visceral adiposity, metabolic syndrome, and related diseases [54,55]. FSHRs have been shown to be expressed in adipocytes [56]. FSH acts by upregulating FSHR in adipocytes, which promotes increased fat accumulation [56,57]. Testing the effects of a polyclonal FSH antibody on bone mass, Liu et al. observed a sharp reduction of adipose tissue in treated wild type mice [58]. Moreover, FSH is able to induce the “beiging” of white adipocytes, contributing to the uncoupling mechanism of thermogenesis (with increased UCP1-rich beige-like adipose tissue) and leanness. Similar results were obtained in haploinsufficient (FSHR^+/−^) mice [58]. FSH also increases adipocyte volume, playing a role in visceral redistribution of body fat [57,58].

In 2020, Han et al. developed a FSH hormone–vaccine capable of preventing fat accumulation in ovariectomized and intact male and female mice by suppressing adipogenic signaling (PPARγ) and upregulating thermogenesis (Ucp1) [59]. These results suggest that FSH plays an independent role in the regulation of body composition. Specifically, increased fat mass and waist circumference, and reduced lean mass, were associated with higher FSH levels in some studies [37,60]. However, in other clinical trials, no correlation between high FSH levels and body mass index (BMI) was observed in men, and, as such, no definitive conclusion can be drawn on the precise role of FSH in influencing human body composition [38,61,62].

### 3.4. Metabolism

Following the evidence of a higher prevalence of hypercholesterolemia in 400 postmenopausal women with high FSH levels [55], extragonadal FSH signaling was also investigated in relation to its supposed effects on lipid biosynthesis. An epidemiological investigation compared 153 pre-menopausal women to 124 peri-menopausal women and reported a direct, estradiol-independent correlation between FSH and cholesterol levels. Moreover, FSH administration in a peri-menopause mouse model resulted in increased cholesterol levels, and the effect could be reverted by blocking FSH signaling with an anti-FSHβ antibody or by FSHR gene ablation, indicating a direct and independent influence of FSH on hepatic cholesterol synthesis [63].

Considering the high prevalence of metabolic syndrome in hypogonadal men, the role of ADT in promoting metabolic alterations has been investigated [64,65,66]. A recent RCT by Østergren et al. compared subjects who underwent bilateral orchiectomy, with patients treated with GnRH-agonists, thus comparing a state of increased vs. decreased FSH concentrations. Increased fat mass and visceral adipose tissue were found in both groups, but they were significantly higher in the orchiectomy group. Although increased insulin resistance and a worse lipid profile were observed in both groups, there were no differences in blood glucose at baseline and after oral glucose tolerance testing (OGTT), and nor were there any significant differences in fasting serum insulin levels and insulin resistance [67,68]. Furthermore, the mechanism underlying the development of impaired glucose metabolism appeared to be related to the increased fat mass, rather than a direct effect [67]. It has also been suggested that the FSH increase in pre-puberty could be associated with a higher probability of developing metabolic syndrome and higher BMI during pubertal transition, although previous findings do not support these conclusions [69,70]. Finally, the role of FSH in the development of non-alcoholic fatty liver disease remains unknown, and the results of the scientific literature are contradictory [39,71]. These data support the complex mechanisms of FSH action involved in metabolism that deserve further study to explore possible therapeutic applications.

### 3.5. Immune System

FSH appears to play a role in immune function and response. As previously reported, FSHR is expressed on immune cells, particularly in monocytes [24]. It was, therefore, hypothesized that FSH could influence the inflammatory response through the production of some cytokines. Data are reported for interferon-γ (IFN-γ), IL-1β [31,72,73] and IL-6 secreted from lipopolysaccharide (LPS) cultured monocytes [74]. A study on mice also described a role of FSH in stimulating IL-6 production from Sertoli cells [75]. According to some studies on CD4^+^ T cell cultures, isolated from peripheral blood mononuclear cells (PBMCs) of healthy donors, stimulation with FSH does not have pro-inflammatory effects [76,77].

In the only in vitro report on males with hypogonadotropic hypogonadism, gonadotropin incubation significantly attenuated the LPS-stimulated secretion of TNF-α and IL-1β by PBMCs [72].

Clinical studies are scanty and mainly focus on the female context of menopause. In menopausal women, in fact, a different inflammatory structure has been described with increased levels of proinflammatory cytokines, such as TNF-α, IL-6, IL-1β and IFN-γ, in addition to neutrophils and monocytes, potentially recruited to strengthen the regulation of the inflammatory reaction [78,79,80]. This inflammatory condition could be responsible for a lot of diseases which increase in menopause, such as metabolic syndrome, type 2 diabetes, and CV disease [81], and may depend on many factors, including hormones. However, it is not clear whether this is related to estrogen deficiency or gonadotropin increase or both. FSH seems to play an important role in menopause, a condition where its levels are directly correlated with the neutrophil-to-lymphocyte ratio (NLR) [82], and monocyte chemoattractant protein-1 (MCP1) [83], TNF-α and IL-6 [84], and, inversely, with CD4^+^ T-lymphocytes, B-lymphocytes and natural killer (NK) cells [85], compared to control women. A positive correlation of FSH with proinflammatory cytokines, along with no association with percentage changes in estradiol and testosterone levels, is also reported in patients with rheumatoid arthritis (both male and female), which could explain the worsening of symptoms in patients with increased FSH (again, for example, during menopause) [86]. As far as the male population is concerned, the literature is almost non-existent. An increased incidence of autoimmune diseases is reported in subjects affected by KS [87,88,89]. However, whether the autoimmune activation is secondary to the decreased testosterone levels per se, or the increased gonadotropin levels is not yet fully understood. In a recent paper, the assessment of 121 newborns (58 males) during mini-puberty was conducted. No correlation with inflammatory cells and cytokines was found in boys, whereas in girls an inverse correlation of FSH levels with CD4^+^ and a direct correlation with CD8^+^ lymphocytes was reported [90]. Corrales and colleagues examined 16 hypogonadal males (6 hyper-, and 10 hypo-gonadotropic) vs. 18 controls. The results showed an increase in CD16^+^ dendritic cells in hypogonadal patients. An inverse correlation between FSH and CD170^+^ expression on T-reg cells was demonstrated in this population, reflecting the occurrence of degranulation exerted during cell activation against target cells and other antigenic and non-antigenic stimuli [91].

### 3.6. Prostate and Other Cancers

Increasing evidence has linked FSH in the development and progression of prostate cancer, as well as in the development of castration-resistant prostate cancer. Indeed, preclinical studies in chemically castrated mice suggest that FSH exerts a direct role on prostate cell growth [92]. Moreover, the presence of FSH, LH and their respective receptors has been reported in prostate cancer cells [93,94]. FSHR expression was found to be low-to-undetectable in normal prostate tissue and in benign prostatic hyperplasia, and consistently high in prostate cancer tissue [95]. Furthermore, FSHR may be relevant in prostate cancer progression, given its dense expression at the periphery of tumors [95]. One small clinical trial investigated the use of abarelix, a GnRH-antagonist, in prostate cancer patients who developed castration-resistant disease following orchiectomy, allowing a reduction in FSH (<5 mIU/L), and the results supported the hypothesis that depleting FSH may have a therapeutic role in castration-resistant prostate cancers. These data demonstrate that GnRH antagonists may have antineoplastic activity beyond their testosterone-reducing properties [96,97].

Besides prostate adenocarcinomas, Radu et al. first described, in 2010, FSHR expression by endothelial cells in a wide range of tumors, including breast, colon, pancreas, bladder, kidney, lung, liver, stomach, testis, and ovary [98], although these results have been heavily criticized [99]. FSHR expression was also described among tumor metastases arising from six different primary tumors (lung, breast, prostate, colon, kidney, and leiomyosarcoma) suggesting a role in the migration and invasion of cancer cells [100]. FSHR-positive vessels could be the result of the tumoral neo-angiogenesis process, as demonstrated by their presence in the normal tissue immediately adjacent to the tumor [98]. The molecular mechanism underlying FSHR-dependent tumor angiogenesis and vascular remodeling could involve hypoxia-inducible factor 1α and the vascular endothelial growth factor (VEGF) pathway, as observed in ovarian granulosa cells [98,101]. Furthermore, FSH was shown to activate the Gq/11 protein [102], which, in turn, induces VEGFR-2 signaling independently of VEGF [103].

Regarding testicular cancer, overexpression of FSHR has been observed in embryonal carcinomas in comparison to seminomas, the latter showing absent FSHR expression. Conversely, estrogen receptor β (ERβ) expression was absent in embryonal carcinoma and present in seminoma. Altogether, ERβ loss and higher FSHR expression have been associated with an advanced testicular tumor stage [100,104].

## 4. Conclusions

In conclusion, the role of FSH in men extends beyond its traditional role in gonadal function. Current data revealed that FSH has extra-gonadal effects in various tissues and organs throughout the body, including the bones, adipose tissue, CV and immune systems. FSH extra-gonadal effects have significant implications for male health and well-being. The literature reviewed in this paper highlights that FSH, previously considered solely a gonadal hormone, has important physiological and pathophysiological effects beyond its role in spermatogenesis in men. The presence of FSHRs in extra-gonadal tissues suggests that FSH in men may play a regulatory role in functions such as bone metabolism, the reduction of BMD, adipose tissue metabolism, and deposition and r health which increase CV events. Doubts remain about its pro-inflammatory function and increase in cholesterol levels. However, the mechanisms underlying these extra-gonadal effects of FSH are still not fully understood. Furthermore, specifically designed trials are needed to dissect the consequences of altered sex steroid levels (e.g., in hypogonadism) from those arising from increased (or decreased) FSH levels on similarly affected target organs, and to better clarify the impact on, for example, the GH/IGF-1 axis.

Although partly conflicting, due to technical and methodological issues, the results from the aforementioned in vitro and in vivo studies open up the possibility of future novel therapeutic strategies for the treatment of some of the most frequent and invalidating chronic diseases, including osteoporosis, obesity, dyslipidemia and cardiovascular disorders. Further research in this field may uncover novel therapeutic targets and interventions for these conditions and shed light on the complex interplay between FSH and extra-gonadal tissues. On the other hand, these results focus attention on potential side effects during FSH treatment in infertile males. As a matter of fact, the aforementioned studies also highlight hypothetical adverse events during FSH therapy in infertile males. Although, for males, a low dosage is commonly used (75–150 UI three times a week), compared to the more common ovarian stimulation protocols, treatments are usually performed for a long time before achieving satisfactory results on spermatogenesis. Therefore, larger RCTs intended to investigate the potential effects of the course of therapy with FSH are needed.

In conclusion, the expanding knowledge of the extra-gonadal effects of FSH in men underscores the importance of considering FSH as a multifunctional hormone with broader physiological significance beyond its role in gonadal function. Therefore, future, and more specific, research is needed to unravel the intricacies of FSH actions in different tissues in order to understand whether stimulation and/or blocking of the receptors may have therapeutic potential and also to investigate any adverse events in infertile males to ensure the therapy is administered safely.

## Data Availability

Not applicable.

## References

[1] Spaziani M., Tarantino C., Tahani N., Gianfrilli D., Sbardella E., Lenzi A., Radicioni A.F. (2021). Hypothalamo-Pituitary axis and puberty. Mol. Cell. Endocrinol..

[2] Bhartiya D., Patel H. (2021). An overview of FSH-FSHR biology and explaining the existing conundrums. J. Ovarian Res..

[3] Recchia K., Jorge A.S., Pessoa L.V.F., Botigelli R.C., Zugaib V.C., de Souza A.F., Martins D.D.S., Ambrosio C.E., Bressan F.F., Pieri N.C.G. (2021). Actions and Roles of FSH in Germinative Cells. Int. J. Mol. Sci..

[4] Simoni M., Gromoll J., Nieschlag E. (1997). The follicle-stimulating hormone receptor: Biochemistry, molecular biology, physiology, and pathophysiology. Endocr. Rev..

[5] Bonfil D., Chuderland D., Kraus S., Shahbazian D., Friedberg I., Seger R., Naor Z. (2004). Extracellular signal-regulated kinase, Jun N-terminal kinase, p38, and c-Src are involved in gonadotropin-releasing hormone-stimulated activity of the glycoprotein hormone follicle-stimulating hormone beta-subunit promoter. Endocrinology.

[6] Rannikko A., Penttila T.L., Zhang F.P., Toppari J., Parvinen M., Huhtaniemi I. (1996). Stage-specific expression of the FSH receptor gene in the prepubertal and adult rat seminiferous epithelium. J. Endocrinol..

[7] Colpi G.M., Francavilla S., Haidl G., Link K., Behre H.M., Goulis D.G., Krausz C., Giwercman A. (2018). European Academy of Andrology guideline Management of oligo-astheno-teratozoospermia. Andrology.

[8] Barbonetti A., Calogero A.E., Balercia G., Garolla A., Krausz C., La Vignera S., Lombardo F., Jannini E.A., Maggi M., Lenzi A. (2018). The use of follicle stimulating hormone (FSH) for the treatment of the infertile man: Position statement from the Italian Society of Andrology and Sexual Medicine (SIAMS). J. Endocrinol. Investig..

[9] Chehab M., Madala A., Trussell J.C. (2015). On-label and off-label drugs used in the treatment of male infertility. Fertil. Steril..

[10] Casarini L., Crepieux P., Reiter E., Lazzaretti C., Paradiso E., Rochira V., Brigante G., Santi D., Simoni M. (2020). FSH for the Treatment of Male Infertility. Int. J. Mol. Sci..

[11] Cannon J.G., Kraj B., Sloan G. (2011). Follicle-stimulating hormone promotes RANK expression on human monocytes. Cytokine.

[12] Stilley J.A., Guan R., Duffy D.M., Segaloff D.L. (2014). Signaling through FSH receptors on human umbilical vein endothelial cells promotes angiogenesis. J. Clin. Endocrinol. Metab..

[13] Oduwole O.O., Peltoketo H., Huhtaniemi I.T. (2018). Role of Follicle-Stimulating Hormone in Spermatogenesis. Front. Endocrinol..

[14] Santi D., Crepieux P., Reiter E., Spaggiari G., Brigante G., Casarini L., Rochira V., Simoni M. (2020). Follicle-stimulating Hormone (FSH) Action on Spermatogenesis: A Focus on Physiological and Therapeutic Roles. J. Clin. Med..

[15] Abel M.H., Baker P.J., Charlton H.M., Monteiro A., Verhoeven G., De Gendt K., Guillou F., O’Shaughnessy P.J. (2008). Spermatogenesis and sertoli cell activity in mice lacking sertoli cell receptors for follicle-stimulating hormone and androgen. Endocrinology.

[16] Tenuta M., Carlomagno F., Cangiano B., Kanakis G., Pozza C., Sbardella E., Isidori A.M., Krausz C., Gianfrilli D. (2021). Somatotropic-Testicular Axis: A crosstalk between GH/IGF-I and gonadal hormones during development, transition, and adult age. Andrology.

[17] Welsh M., Saunders P.T., Atanassova N., Sharpe R.M., Smith L.B. (2009). Androgen action via testicular peritubular myoid cells is essential for male fertility. FASEB J..

[18] Huhtaniemi I. (2010). A hormonal contraceptive for men: How close are we?. Prog. Brain Res..

[19] Davies A.G. (1981). Role of FSH in the control of testicular function. Arch. Androl..

[20] Hasenmajer V., Bonaventura I., Minnetti M., Sada V., Sbardella E., Isidori A.M. (2021). Non-Canonical Effects of ACTH: Insights Into Adrenal Insufficiency. Front. Endocrinol..

[21] Khosla S., Oursler M.J., Monroe D.G. (2012). Estrogen and the skeleton. Trends Endocrinol. Metab..

[22] Khosla S., Monroe D.G. (2018). Regulation of Bone Metabolism by Sex Steroids. Cold Spring Harb. Perspect. Med..

[23] Sowers M.R., Zheng H., McConnell D., Nan B., Harlow S., Randolph J.F. (2008). Follicle stimulating hormone and its rate of change in defining menopause transition stages. J. Clin. Endocrinol. Metab..

[24] Robinson L.J., Tourkova I., Wang Y., Sharrow A.C., Landau M.S., Yaroslavskiy B.B., Sun L., Zaidi M., Blair H.C. (2010). FSH-receptor isoforms and FSH-dependent gene transcription in human monocytes and osteoclasts. Biochem. Biophys. Res. Commun..

[25] Sun L., Peng Y., Sharrow A.C., Iqbal J., Zhang Z., Papachristou D.J., Zaidi S., Zhu L.L., Yaroslavskiy B.B., Zhou H. (2006). FSH directly regulates bone mass. Cell.

[26] Gera S., Sant D., Haider S., Korkmaz F., Kuo T.C., Mathew M., Perez-Pena H., Xie H., Chen H., Batista R. (2020). First-in-class humanized FSH blocking antibody targets bone and fat. Proc. Natl. Acad. Sci. USA.

[27] Zhu L.L., Blair H., Cao J., Yuen T., Latif R., Guo L., Tourkova I.L., Li J., Davies T.F., Sun L. (2012). Blocking antibody to the beta-subunit of FSH prevents bone loss by inhibiting bone resorption and stimulating bone synthesis. Proc. Natl. Acad. Sci. USA.

[28] Ji Y., Liu P., Yuen T., Haider S., He J., Romero R., Chen H., Bloch M., Kim S.M., Lizneva D. (2018). Epitope-specific monoclonal antibodies to FSHbeta increase bone mass. Proc. Natl. Acad. Sci. USA.

[29] Geng W., Yan X., Du H., Cui J., Li L., Chen F. (2013). Immunization with FSHbeta fusion protein antigen prevents bone loss in a rat ovariectomy-induced osteoporosis model. Biochem. Biophys. Res. Commun..

[30] Iqbal J., Sun L., Kumar T.R., Blair H.C., Zaidi M. (2006). Follicle-stimulating hormone stimulates TNF production from immune cells to enhance osteoblast and osteoclast formation. Proc. Natl. Acad. Sci. USA.

[31] Cannon J.G., Cortez-Cooper M., Meaders E., Stallings J., Haddow S., Kraj B., Sloan G., Mulloy A. (2010). Follicle-stimulating hormone, interleukin-1, and bone density in adult women. Am. J. Physiol. Regul. Integr. Comp. Physiol..

[32] Ritter V., Thuering B., Saint Mezard P., Luong-Nguyen N.H., Seltenmeyer Y., Junker U., Fournier B., Susa M., Morvan F. (2008). Follicle-stimulating hormone does not impact male bone mass in vivo or human male osteoclasts in vitro. Calcif. Tissue Int..

[33] Giovanelli L., Quinton R., Cangiano B., Colombo S., Persani L., Bonomi M., Chiodini I. (2022). FSH and bone: Comparison between males with central versus primary hypogonadism. Front. Endocrinol..

[34] Ferlin A., Schipilliti M., Vinanzi C., Garolla A., Di Mambro A., Selice R., Lenzi A., Foresta C. (2011). Bone mass in subjects with Klinefelter syndrome: Role of testosterone levels and androgen receptor gene CAG polymorphism. J. Clin. Endocrinol. Metab..

[35] Tahani N., Nieddu L., Prossomariti G., Spaziani M., Granato S., Carlomagno F., Anzuini A., Lenzi A., Radicioni A.F., Romagnoli E. (2018). Long-term effect of testosterone replacement therapy on bone in hypogonadal men with Klinefelter Syndrome. Endocrine.

[36] Karim N., MacDonald D., Dolan A.L., Fogelman I., Wierzbicki A.S., Hampson G. (2008). The relationship between gonadotrophins, gonadal hormones and bone mass in men. Clin. Endocrinol..

[37] Sowers M., Zheng H., Tomey K., Karvonen-Gutierrez C., Jannausch M., Li X., Yosef M., Symons J. (2007). Changes in body composition in women over six years at midlife: Ovarian and chronological aging. J. Clin. Endocrinol. Metab..

[38] Sowers M.R., Finkelstein J.S., Ettinger B., Bondarenko I., Neer R.M., Cauley J.A., Sherman S., Greendale G.A. (2003). Study of Women’s Health Across the N. The association of endogenous hormone concentrations and bone mineral density measures in pre- and perimenopausal women of four ethnic groups: SWAN. Osteoporos. Int..

[39] Wang N., Li Q., Han B., Chen Y., Zhu C., Chen Y., Xia F., Lu M., Meng Y., Guo Y. (2016). Follicle-stimulating hormone is associated with non-alcoholic fatty liver disease in Chinese women over 55 years old. J. Gastroenterol. Hepatol..

[40] Antonio L., Priskorn L., Olesen I.A., Petersen J.H., Vanderschueren D., Jorgensen N. (2020). High serum FSH is not a risk factor for low bone mineral density in infertile men. Bone.

[41] Antonio L., Priskorn L., Nordkap L., Bang A.K., Jensen T.K., Skakkebaek N.E., Petersen J.H., Vanderschueren D., Jorgensen N. (2020). Bone mineral density is preserved in men with idiopathic infertility. Andrology.

[42] Uihlein A.V., Finkelstein J.S., Lee H., Leder B.Z. (2014). FSH suppression does not affect bone turnover in eugonadal men. J. Clin. Endocrinol. Metab..

[43] Chen H., Guo J.H., Lu Y.C., Ding G.L., Yu M.K., Tsang L.L., Fok K.L., Liu X.M., Zhang X.H., Chung Y.W. (2012). Impaired CFTR-dependent amplification of FSH-stimulated estrogen production in cystic fibrosis and PCOS. J. Clin. Endocrinol. Metab..

[44] Robinson C.A., Hofer M., Benden C., Schmid C. (2019). Evaluation of bone disease in patients with cystic fibrosis and end-stage lung disease. J. Bras. Pneumol..

[45] El Khoudary S.R., Wildman R.P., Matthews K., Thurston R.C., Bromberger J.T., Sutton-Tyrrell K. (2012). Endogenous sex hormones impact the progression of subclinical atherosclerosis in women during the menopausal transition. Atherosclerosis.

[46] El Khoudary S.R., Santoro N., Chen H.Y., Tepper P.G., Brooks M.M., Thurston R.C., Janssen I., Harlow S.D., Barinas-Mitchell E., Selzer F. (2016). Trajectories of estradiol and follicle-stimulating hormone over the menopause transition and early markers of atherosclerosis after menopause. Eur. J. Prev. Cardiol..

[47] Munir J.A., Wu H., Bauer K., Bindeman J., Byrd C., Feuerstein I.M., Villines T.C., Taylor A.J. (2012). The perimenopausal atherosclerosis transition: Relationships between calcified and noncalcified coronary, aortic, and carotid atherosclerosis and risk factors and hormone levels. Menopause.

[48] Hopmans S.N., Duivenvoorden W.C., Werstuck G.H., Klotz L., Pinthus J.H. (2014). GnRH antagonist associates with less adiposity and reduced characteristics of metabolic syndrome and atherosclerosis compared with orchiectomy and GnRH agonist in a preclinical mouse model. Urol. Oncol..

[49] Han J.L., Song Y.X., Yao W.J., Zhou J., Du Y., Xu T. (2022). Follicle-Stimulating Hormone Provokes Macrophages to Secrete IL-1beta Contributing to Atherosclerosis Progression. J. Immunol..

[50] Albertsen P.C., Klotz L., Tombal B., Grady J., Olesen T.K., Nilsson J. (2014). Cardiovascular morbidity associated with gonadotropin releasing hormone agonists and an antagonist. Eur. Urol..

[51] Crawford E.D., Schally A.V., Pinthus J.H., Block N.L., Rick F.G., Garnick M.B., Eckel R.H., Keane T.E., Shore N.D., Dahdal D.N. (2017). The potential role of follicle-stimulating hormone in the cardiovascular, metabolic, skeletal, and cognitive effects associated with androgen deprivation therapy. Urol. Oncol..

[52] Haring R., Teng Z., Xanthakis V., Coviello A., Sullivan L., Bhasin S., Murabito J.M., Wallaschofski H., Vasan R.S. (2013). Association of sex steroids, gonadotrophins, and their trajectories with clinical cardiovascular disease and all-cause mortality in elderly men from the Framingham Heart Study. Clin. Endocrinol..

[53] Kourbanhoussen K., Joncas F.H., Wallis C.J.D., Hovington H., Dagenais F., Fradet Y., Guillemette C., Lacombe L., Toren P. (2022). Follicle-stimulating hormone (FSH) levels prior to prostatectomy are not related to long-term oncologic or cardiovascular outcomes for men with prostate cancer. Asian J. Androl..

[54] Stefanska A., Sypniewska G., Ponikowska I., Cwiklinska-Jurkowska M. (2012). Association of follicle-stimulating hormone and sex hormone binding globulin with the metabolic syndrome in postmenopausal women. Clin. Biochem..

[55] Song Y., Wang E.S., Xing L.L., Shi S., Qu F., Zhang D., Li J.Y., Shu J., Meng Y., Sheng J.Z. (2016). Follicle-Stimulating Hormone Induces Postmenopausal Dyslipidemia Through Inhibiting Hepatic Cholesterol Metabolism. J. Clin. Endocrinol. Metab..

[56] Cui H., Zhao G., Liu R., Zheng M., Chen J., Wen J. (2012). FSH stimulates lipid biosynthesis in chicken adipose tissue by upregulating the expression of its receptor FSHR. J. Lipid Res..

[57] Liu X.M., Chan H.C., Ding G.L., Cai J., Song Y., Wang T.T., Zhang D., Chen H., Yu M.K., Wu Y.T. (2015). FSH regulates fat accumulation and redistribution in aging through the Galphai/Ca(2+)/CREB pathway. Aging Cell..

[58] Liu P., Ji Y., Yuen T., Rendina-Ruedy E., DeMambro V.E., Dhawan S., Abu-Amer W., Izadmehr S., Zhou B., Shin A.C. (2017). Blocking FSH induces thermogenic adipose tissue and reduces body fat. Nature.

[59] Han X., Guan Z., Xu M., Zhang Y., Yao H., Meng F., Zhuo Y., Yu G., Cao X., Du X. (2020). A novel follicle-stimulating hormone vaccine for controlling fat accumulation. Theriogenology.

[60] Seth B., Arora S., Singh R. (2013). Association of obesity with hormonal imbalance in infertility: A cross-sectional study in north Indian women. Indian. J. Clin. Biochem..

[61] Bieniek J.M., Kashanian J.A., Deibert C.M., Grober E.D., Lo K.C., Brannigan R.E., Sandlow J.I., Jarvi K.A. (2016). Influence of increasing body mass index on semen and reproductive hormonal parameters in a multi-institutional cohort of subfertile men. Fertil. Steril..

[62] Yamacake K.G., Cocuzza M., Torricelli F.C., Tiseo B.C., Frati R., Freire G.C., Antunes A.A., Srougi M. (2016). Impact of body mass index, age and varicocele on reproductive hormone profile from elderly men. Int. Braz. J. Urol..

[63] Guo Y., Zhao M., Bo T., Ma S., Yuan Z., Chen W., He Z., Hou X., Liu J., Zhang Z. (2019). Blocking FSH inhibits hepatic cholesterol biosynthesis and reduces serum cholesterol. Cell Res..

[64] Keating N.L., O’Malley A.J., Freedland S.J., Smith M.R. (2013). Does comorbidity influence the risk of myocardial infarction or diabetes during androgen-deprivation therapy for prostate cancer?. Eur. Urol..

[65] Basaria S., Muller D.C., Carducci M.A., Egan J., Dobs A.S. (2006). Hyperglycemia and insulin resistance in men with prostate carcinoma who receive androgen-deprivation therapy. Cancer.

[66] Morote J., Gomez-Caamano A., Alvarez-Ossorio J.L., Pesqueira D., Tabernero A., Gomez Veiga F., Lorente J.A., Porras M., Lobato J.J., Ribal M.J. (2015). The metabolic syndrome and its components in patients with prostate cancer on androgen deprivation therapy. J. Urol..

[67] Ostergren P.B., Kistorp C., Fode M., Bennedbaek F.N., Faber J., Sonksen J. (2019). Metabolic consequences of gonadotropin-releasing hormone agonists vs orchiectomy: A randomized clinical study. BJU Int..

[68] Cheung A.S., Hoermann R., Dupuis P., Joon D.L., Zajac J.D., Grossmann M. (2016). Relationships between insulin resistance and frailty with body composition and testosterone in men undergoing androgen deprivation therapy for prostate cancer. Eur. J. Endocrinol..

[69] Vandewalle S., Taes Y., Fiers T., Van Helvoirt M., Debode P., Herregods N., Ernst C., Van Caenegem E., Roggen I., Verhelle F. (2014). Sex steroids in relation to sexual and skeletal maturation in obese male adolescents. J. Clin. Endocrinol. Metab..

[70] Aydin B.K., Stenlid R., Ciba I., Cerenius S.Y., Dahlbom M., Bergsten P., Nergardh R., Forslund A. (2022). High levels of FSH before puberty are associated with increased risk of metabolic syndrome during pubertal transition. Pediatr. Obes..

[71] Zhu Y., Xu J., Zhang X., Ke Y., Fu G., Guo Q. (2021). A low follicle-stimulating hormone level is a protective factor for non-alcoholic fatty liver disease in older men aged over 80. BMC Geriatr..

[72] Musabak U., Bolu E., Ozata M., Oktenli C., Sengul A., Inal A., Yesilova Z., Kilciler G., Ozdemir I.C., Kocar I.H. (2003). Gonadotropin treatment restores in vitro interleukin-1beta and tumour necrosis factor-alpha production by stimulated peripheral blood mononuclear cells from patients with idiopathic hypogonadotropic hypogonadism. Clin. Exp. Immunol..

[73] Yousefi S., Karamlou K., Vaziri N., Carandang G., Ocariz J., Cesario T. (1993). The effect of gonadotropins on the production of human interferon-gamma by mononuclear cells. J. Interferon Res..

[74] Komorowski J., Stepien H. (1994). FSH and LH induce interleukin-6 (IL-6) release from human peripheral blood monocytes cultures in vitro. A dose-response study. Horm. Metab. Res..

[75] Syed V., Gerard N., Kaipia A., Bardin C.W., Parvinen M., Jegou B. (1993). Identification, ontogeny, and regulation of an interleukin-6-like factor in the rat seminiferous tubule. Endocrinology.

[76] Carbone F., Procaccini C., De Rosa V., Alviggi C., De Placido G., Kramer D., Longobardi S., Matarese G. (2010). Divergent immunomodulatory effects of recombinant and urinary-derived FSH, LH, and hCG on human CD4+ T cells. J. Reprod. Immunol..

[77] Biffoni M., Marcucci I., Ythier A., Eshkol A. (1998). Effects of urinary gonadotrophin preparations on human in-vitro immune function. Hum. Reprod..

[78] Cenci S., Toraldo G., Weitzmann M.N., Roggia C., Gao Y., Qian W.P., Sierra O., Pacifici R. (2003). Estrogen deficiency induces bone loss by increasing T cell proliferation and lifespan through IFN-gamma-induced class II transactivator. Proc. Natl. Acad. Sci. USA.

[79] Stubelius A., Andersson A., Islander U., Carlsten H. (2017). Ovarian hormones in innate inflammation. Immunobiology.

[80] Tyagi A.M., Srivastava K., Kureel J., Kumar A., Raghuvanshi A., Yadav D., Maurya R., Goel A., Singh D. (2012). Premature T cell senescence in Ovx mice is inhibited by repletion of estrogen and medicarpin: A possible mechanism for alleviating bone loss. Osteoporos. Int..

[81] Atsma F., Bartelink M.L., Grobbee D.E., van der Schouw Y.T. (2006). Postmenopausal status and early menopause as independent risk factors for cardiovascular disease: A meta-analysis. Menopause.

[82] Ilhan G., Atmaca F.F.V., Altan E., Zebitay A.G., Sozen H., Akyol H., Kurek Eken M. (2016). Evaluation of Neutrophil-Lymphocyte Ratio, Platelet-Lymphocyte Ratio and Red Blood Cell Distribution Width-Platelet Ratio for Diagnosis of Premature Ovarian Insufficiency. J. Fam. Reprod. Health.

[83] Tani A., Yasui T., Matsui S., Kato T., Kunimi K., Tsuchiya N., Yuzurihara M., Kase Y., Irahara M. (2013). Different circulating levels of monocyte chemoattractant protein-1 and interleukin-8 during the menopausal transition. Cytokine.

[84] Abildgaard J., Tingstedt J., Zhao Y., Hartling H.J., Pedersen A.T., Lindegaard B., Dam Nielsen S. (2020). Increased systemic inflammation and altered distribution of T-cell subsets in postmenopausal women. PLoS ONE.

[85] Giglio T., Imro M.A., Filaci G., Scudeletti M., Puppo F., De Cecco L., Indiveri F., Costantini S. (1994). Immune cell circulating subsets are affected by gonadal function. Life Sci..

[86] Kass A.S., Lea T.E., Torjesen P.A., Gulseth H.C., Forre O.T. (2010). The association of luteinizing hormone and follicle-stimulating hormone with cytokines and markers of disease activity in rheumatoid arthritis: A case-control study. Scand. J. Rheumatol..

[87] Sawalha A.H., Harley J.B., Scofield R.H. (2009). Autoimmunity and Klinefelter’s syndrome: When men have two X chromosomes. J. Autoimmun..

[88] Seminog O.O., Seminog A.B., Yeates D., Goldacre M.J. (2015). Associations between Klinefelter’s syndrome and autoimmune diseases: English national record linkage studies. Autoimmunity.

[89] Rovensky J., Imrich R., Lazurova I., Payer J. (2010). Rheumatic diseases and Klinefelter’s syndrome. Ann. NY. Acad. Sci..

[90] Karaoglan M., Nacarkahya G. (2021). Immunological interpretation of minipuberty: Minipuberty as the driving force of sexual dimorphism in the immune response. Clin. Endocrinol..

[91] Corrales J.J., Almeida M., Cordero M., Martin-Martin L., Mendez C., Miralles J.M., Orfao A. (2012). Enhanced immunological response by dendritic cells in male hypogonadism. Eur. J. Clin. Investig..

[92] Deiktakis E.E., Ieronymaki E., Zaren P., Hagsund A., Wirestrand E., Malm J., Tsatsanis C., Huhtaniemi I.T., Giwercman A., Giwercman Y.L. (2022). Impact of add-back FSH on human and mouse prostate following gonadotropin ablation by GnRH antagonist treatment. Endocr. Connect..

[93] Dirnhofer S., Berger C., Hermann M., Steiner G., Madersbacher S., Berger P. (1998). Coexpression of gonadotropic hormones and their corresponding FSH- and LH/CG-receptors in the human prostate. Prostate.

[94] Ben-Josef E., Yang S.Y., Ji T.H., Bidart J.M., Garde S.V., Chopra D.P., Porter A.T., Tang D.G. (1999). Hormone-refractory prostate cancer cells express functional follicle-stimulating hormone receptor (FSHR). J. Urol..

[95] Mariani S., Salvatori L., Basciani S., Arizzi M., Franco G., Petrangeli E., Spera G., Gnessi L. (2006). Expression and cellular localization of follicle-stimulating hormone receptor in normal human prostate, benign prostatic hyperplasia and prostate cancer. J. Urol..

[96] Gartrell B.A., Tsao C.K., Galsky M.D. (2013). The follicle-stimulating hormone receptor: A novel target in genitourinary malignancies. Urol. Oncol..

[97] Beer T.M., Garzotto M., Eilers K.M., Lemmon D., Wersinger E.M. (2004). Targeting FSH in androgen-independent prostate cancer: Abarelix for prostate cancer progressing after orchiectomy. Urology.

[98] Radu A., Pichon C., Camparo P., Antoine M., Allory Y., Couvelard A., Fromont G., Hai M.T., Ghinea N. (2010). Expression of follicle-stimulating hormone receptor in tumor blood vessels. N. Engl. J. Med..

[99] Chrusciel M., Ponikwicka-Tyszko D., Wolczynski S., Huhtaniemi I., Rahman N.A. (2019). Extragonadal FSHR Expression and Function-Is It Real?. Front. Endocrinol..

[100] Siraj A., Desestret V., Antoine M., Fromont G., Huerre M., Sanson M., Camparo P., Pichon C., Planeix F., Gonin J. (2013). Expression of follicle-stimulating hormone receptor by the vascular endothelium in tumor metastases. BMC Cancer.

[101] Alam H., Weck J., Maizels E., Park Y., Lee E.J., Ashcroft M., Hunzicker-Dunn M. (2009). Role of the phosphatidylinositol-3-kinase and extracellular regulated kinase pathways in the induction of hypoxia-inducible factor (HIF)-1 activity and the HIF-1 target vascular endothelial growth factor in ovarian granulosa cells in response to follicle-stimulating hormone. Endocrinology.

[102] Castro-Fernandez C., Maya-Nunez G., Mendez J.P. (2004). Regulation of follicle-stimulating and luteinizing hormone receptor signaling by. Endocrine.

[103] Zeng H., Zhao D., Yang S., Datta K., Mukhopadhyay D. (2003). Heterotrimeric G alpha q/G alpha 11 proteins function upstream of vascular endothelial growth factor (VEGF) receptor-2 (KDR) phosphorylation in vascular permeability factor/VEGF signaling. J. Biol. Chem..

[104] Panza S., Giordano F., De Rose D., Panno M.L., De Amicis F., Santoro M., Malivindi R., Rago V., Aquila S. (2020). FSH-R Human Early Male Genital Tract, Testicular Tumors and Sperm: Its Involvement in Testicular Disorders. Life.

